# Evidence already exists for motor system reorganization in CRPS

**DOI:** 10.1080/24740527.2017.1422976

**Published:** 2018-01-30

**Authors:** Shabbir Hussain I. Merchant

**Affiliations:** Human Motor Control Section, National Institute of Neurological Disorders and Stroke, National Institutes of Health, Bethesda, Maryland

**Keywords:** complex regional pain syndrome, transcranial magnetic stimulation, motor system, corticospinal tracts, graded motor imagery

## Abstract

Complex regional pain syndrome (CRPS) is a disabling condition that is usually preceded by trauma or surgical procedure. Involvement of the motor system is a well-known phenomenon in CRPS, though the pathophysiologic mechanisms of motor system affliction in CRPS are poorly understood. Graded motor imagery (GMI) has been proposed to be one of the therapeutic interventions to help improve pain and other disabling symptoms associated with CRPS, though the benefits noted are modest and inconsistent. The neurophysiological mechanisms implicated in motor imagery are intended to target the aberrant prefrontal and sensorimotor integration areas, which may potentially help restore the aberrant cortical plasticity in CRPS. Detailed well-controlled experiments using insights from the existing body of literature on motor system reorganization in CRPS are required to better understand this complicated disorder. Attempts to gain pathophysiologic insights about complicated disorders like CRPS based on case reports with poorly performed and uncontrolled interventions are misguided.

## RÉSUMÉ

Le syndrome douleureux régional complexe est une affection invalidante habituellement précédée d’un traumatisme ou d’une intervention chirurgicale. L’implication du système moteur dans le SDRC est un phénomène bien connu, malgré le fait que les mécanismes pathophysiologiques qui l’affectent soient mal compris. L’imagerie motrice progressive (IMP) a été proposée en tant que l’une des interventions théraeutiques pouvant aider à améliorer la douleur et d’autres symptômes invalidants associés au SDRC, bien que les effets bénéfiques observés soient modestes et contradictoires. Les mécanismes neurophysiologiques impliqués dans l’imagerie motrice sont destinés à cibler l’aire préfrontale et l’aire d’intégration sensorimotrice anormales qui peuvent potentiellement aider à rétablir la plasticité corticale dans le SDRC. Des expériences approfondies bien contrôlées fondées sur les connaissances que l’on retrouve dans la littérature existante en ce qui concerne la réorganisation du système moteur dans le SDRC sont nécessaires afin de mieux comprendre ce trouble compliqué. Les tentatives pour améliorer les connaissances pathophsyiologiques concernant des troubles compliqués comme le SDRC qui sont fondées sur des études de cas et des interventions effectuées de manière médiocre et non contrôlées sont malavisées.

Complex regional pain syndrome (CRPS) was first described by Weir Mitchell as a vasomotor neurosis and he has been credited for first using the term *causalgia* in his writing to describe the symptoms associated with this rather elusive disorder.^1–5^ CRPS still remains a poorly understood and disabling condition.^6–8^ It is usually preceded by minor to severe trauma or surgical procedure.^9–11^ Involvement of the motor system is a well-known phenomenon in CRPS, though the pathophysiologic mechanisms involved are poorly understood.^12–17^ Movement disorders in CRPS have been variously described as loss of voluntary control or failure to initiate movement, weakness, bradykinesia, dystonia, myoclonus, spasm, and tremor.^18–20^ The current literature, which includes studies based on noninvasive brain stimulation techniques like transcranial magnetic stimulation (TMS), support increased cortical excitability in CRPS, and normalizing these cortical aberrancies can potentially help improve the motor performance in CRPS.^21–23^

In the present issue of the *Canadian Journal of Pain*, Harvey et al. describe the case of a 58-year-old woman who developed CRPS after sustaining a radial fracture.^24^ They treated her with graded motor imagery and noted some improvements in her pain without any significant objective improvement in the motor disability. They additionally performed TMS during the follow-up visits and noted a serial increase in delta scores based on motor evoked potential (MEP) amplitudes and interpreted that as being evidence for motor system reorganization. The current case, which occurred after a radial fracture was classified as CRPS type I; however, the authors do not report any findings of electromyography (EMG)/nerve conduction studies, which would be essential for this diagnosis, especially in the setting of a fracture. Absence of baseline and follow-up nerve conduction studies to rule out the possibility of any axonotmesis or neurapraxia makes the interpretation of the findings of the current case report difficult. The authors correctly noted many limitations in their performance and interpretation of the TMS experiments. To note a few, they used delta scores derived using resting motor threshold (RMT) as a surrogate for corticospinal strength, which is incorrect. They did not perform an input-output (IO) curve, which involves giving TMS pulses of varying strengths in a random order to measure the MEPs at the different stimulation intensities. Additionally, using the same swim cap to locate the hotspot for follow-up visits is an erroneous technique. Though using swim caps may be reasonable technique for a single session, it is certainly not appropriate for multiple follow-up visits, and imaging-based guidance should have been used to ensure stimulation of the same cortical site.

The authors suggested improvements noted in delta scores that are based on RMT obtained at follow-up visits as suggestive of strengthening of corticospinal projections. RMT is increased by drugs blocking the voltage-gated sodium channels and decreased by drugs that enhance non-N-methly-D-aspartate (NMDA)-mediated glutamatergic transmission.^25–27^ There is no clear evidence that any of the interventions performed by the authors influence either of these mechanisms. Mechanisms of graded motor imagery, though poorly understood, implicate influencing the prefrontal and sensorimotor integration cortices.^28–30^ Studying the influence of a complicated therapeutic intervention using measures based on RMT does not have any clear rationale. The authors claim improvements in the MEP recorded as evidence of strengthening of corticospinal projections; however, the clinical improvements in strength are clearly lacking.

The findings of the current case report can be best interpreted as improved recording of the surface EMG as the vasomotor and sudomotor changes associated with CRPS improved. Though the authors noted nonsignificant changes in the edema, the peripheral changes were not clearly monitored objectively at follow-up, to be certain. Though the changes related to edema can affect the MEPs at both 110% and 130%, the variability in the MEPs is higher at intensities between 120% and 140% RMT and, as a result, the delta scores may be further falsely inflated.^31^ Another possible explanation could be the resolution of the nerve damage, which may have occurred due to the fracture and would follow a similar timeline for improvement.^32^ Improvement in the strength of corticospinal projections is an unlikely explanation for the findings noted in the current report.

Evidence does exist for motor system reorganization in CRPS and I reference some of the current literature on the subject in this write-up. The efforts by the authors to find objective measures to corroborate improvements in motor function are commendable. However, interpretation of their findings as evidence for motor system reorganization is misguided in my opinion.

## Disclosure of Interest

Shabbir Hussain Merchant has no relevant conflict of interest to declare related to this publication.
